# Viroid-like “obelisk” agents are widespread in the ocean and exceed the abundance of RNA viruses in the prokaryotic fraction

**DOI:** 10.1093/ismejo/wraf033

**Published:** 2025-02-25

**Authors:** Javier López-Simón, Marcos de la Peña, Manuel Martínez-García

**Affiliations:** Instituto Multidisciplinar para el Estudio del Medio Ramon Margalef, Parque Científico, Edificio Nuevos Institutos, University of Alicante, Ap-Correos 99, San Vicente del Raspeig E-03690, Spain; Departament of Physiology, Genetics, and Microbiology, University of Alicante, Carretera de San Vicente s/n, San Vicente del Raspeig 03080, Spain; Instituto de Biología Molecular y Celular de Plantas, Universidad Politécnica de Valencia-CSIC, Calle Ingeniero Fausto Elio s/n, Valencia 46022, Spain; Instituto Multidisciplinar para el Estudio del Medio Ramon Margalef, Parque Científico, Edificio Nuevos Institutos, University of Alicante, Ap-Correos 99, San Vicente del Raspeig E-03690, Spain; Departament of Physiology, Genetics, and Microbiology, University of Alicante, Carretera de San Vicente s/n, San Vicente del Raspeig 03080, Spain

**Keywords:** RNA viroids, obelisk, RNA viruses, marine, ocean, bacteria, prokaryotes, Oblins

## Abstract

“Obelisks” are recently discovered ribonucleic acid (RNA) viroid-like elements present in diverse environments with no phylogenetic similarity to any known biological agent. obelisks were first identified in the human gut and in a commensal bacterium acting as a replicative host. They have a circular ∼1 kb RNA genome, rod-like secondary structures, and the encoding of a protein superfamily called “Oblins”. We performed a large-scale search of obelisks in the ocean using the Pebblescout program and the transcriptomic Sequence Archive Read databases, revealing the biogeography and abundance of these viroid-like RNA elements. We detected 55 obelisk genomes resulting in 35 marine clusters at the species level. These obelisks were detected in the prokaryotic fraction and to a lesser extent in the eukaryotic fraction, and distributed across all the oceans from surface to mesopelagic including the Arctic, and even in the coldest seawater of Earth beneath the Antarctic Ross Ice Shelf. The obelisk hallmark protein Oblin-1 confirmed by 3D models was found in various marine samples. Some of the detected marine obelisks harbor hammerhead self-cleaving ribozymes in both polarities. In the prokaryotic, but not the eukaryotic, fraction of the *Tara* Ocean dataset, relative abundance of obelisks calculated by transcriptomic fragment recruitment indicated that they are abundant in marine samples, reaching or even exceeding the relative abundance of the previously discovered uncultured RNA viruses. In conclusion, obelisks are abundant and widespread viroid-like elements that should be included in ocean biogeochemical models.

##  

Ribonucleic acid (RNA) viruses are agents that carry RNA as their genetic material and replicate using their own RNA-dependent RNA polymerase (RdRp), a universal “hallmark” gene of RNA viruses [1]. Historically, these viruses have been less studied albeit in recent years, interest in RNA viruses has increased, and new RNA viruses are constantly being discovered from different metatranscriptome datasets [2–6]. Not to mention the interest in the RNA world hypothesis, which proposes that on a primitive Earth, RNA was the primary living substance [7].

**Figure 1:**
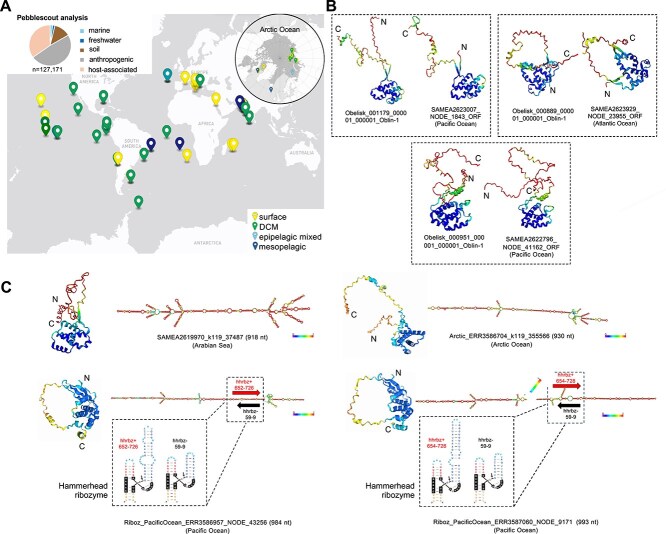
Obelisks RNA viroid-like elements are widespread in oceanic environments. (A) World map showing the locations of the analyzed *Tara* metatranscriptomes where obelisks were detected (>70% nucleotide identity and > 50% query coverage) using our sequence homology search strategy in surface (3-7 m depth), DCM layer (28-180 m depth), epipelagic mixed layer (21-107 m depth), and mesopelagic (262-812 m) samples. Upper left insert panel shows Pebblescout analysis identifying >100 000 hits with >80% of PBS score in different environmental samples using obelisks as query. (B) Predicted tertiary structure (ColabFold v1.5.5, AlphaFold2 using MMseqs2) of the Oblin-1 protein of some obelisks. Three pairs of Oblins are shown and, in each pair, the predicted Oblin-1 from the marine samples and the Oblin-1 of the formerly described obelisks are displayed. A clear structural similarity is observed. Structure of Oblin-1 is defined as a N-terminal (indicated as “N”) globule that includes a bundle of alpha helices, capped with a beta sheet clasp and connected by a region containing conserved domain-A, which lacks a good tertiary structure prediction and ends with a C-terminal (indicated as “C”) alpha helix. Colors indicate a measure of confidence in the local structure (*pLDDT*), going from red (low confidence) to blue (very high confidence). (c) Four examples of detected obelisks that do not fit into any of the previously described obelisk clusters, suggesting an autochthonous diversity of marine obelisks. For each one, two hallmark characteristics of obelisks are represented: The presence of Oblin-1 (predicted tertiary structure) and the rod-shaped secondary structure (predicted by RNAfold). In the bottom row, there are two examples of obelisks with an obelisk variant hhrbz (hammerhead ribozyme) detected in the Pacific Ocean. Within the boxed area, we can find the region where the ribozyme is located and its structure.

Viroids are non-coding, sub-viral, single-stranded circular RNA molecules with a broad presence in diverse transcriptomes. They replicate and infect commonly plants, and are thought to be survivors of the RNA world [8]. Recently, some hybrids of RNA viruses and viroid-like elements have been discovered, such as fungal ambiviruses and mitoviruses, which are large infectious RNAs with circular genomes that undergo rolling circle replication and encode their own RdRp and self-cleaving ribozymes, suggesting a co-evolutionary history between RNA viruses and subviral elements [9, 10]. More recently, obelisks were described as unrecognized viroid-like entities identified in metatranscriptomic data from the human gut [11]. They form a distinct phylogenetic group with no similarity to any known biological agent and share several characteristics, including apparently circular RNA of about 1 kb, rod-like secondary structures encompassing the entire genome, and open reading frames that encode for a protein superfamily called “Oblin”. The Oblin-1 protein appears to have an RNA-binding function, based on the prediction of the tertiary structure and the presence of a specific domain enriched with positively charged residues that could bind anions, such as RNA. The Oblin-2 has a potential function as a homo-multimer, or as a binding partner to other host leucine zippers [11]. Obelisks are prevalent in human microbiome metatranscriptomes and a specific obelisk population harbored by a laboratory strain of *Streptococcus sanguinis* has also been identified. Additionally, a subset of obelisks is identified to code for specific variants of the type-III hammerhead self-cleaving ribozyme in both polarities, suggesting a viroid-like replication system [11]. However, whether the presence of these unrecognized obelisk RNA viroid-like elements in marine ecosystems is merely anecdotal remains an open question, which is important for building accurate ecological models. This study provides biological insights into the ecogenomics, biogeography, and abundance of obelisks in the ocean.

Previously, a pioneer study built a conservative database of 7202 obelisk genomes that were clustered into 1744 groups using 80% nucleotide identity threshold, and later subclustered at 95% nucleotide identity as a proxy of species-level [11]. In our study, we detected the presence of some of these previously described obelisks in different marine samples by using a massive search with Pebblescout program, which allows very fast and efficient searches for subjects in petabase-scale nucleotide databases [12]. We ran the obelisk database against the entire metagenomic database of Sequence Read Archive, which includes all metagenomic and metatranscriptomic studies released before the end of 2021. Our analysis identified more than 100 000 hits with >80% of PBS score (Fig. 1A, Supplementary Table 1) in numerous samples including marine ecosystems, such as those collected from the *Tara* Oceans project [13]. We detected obelisks in 51 out of 159 *Tara* Ocean prokaryotic metatranscriptomes (e-value <0.00001, nucleotide identity >70%, query coverage >50%) spread across all oceans and depths including surface samples (depth 3–7 m), epipelagic mixed layer samples (depth 21–107 m), deep chlorophyll maximum layer samples (depth 28–180 m), and even in mesopelagic layers (depth 262–812 m; Fig. 1A; Supplementary Tables 2 and 3).

In total, we discovered 39 obelisk sequences in addition to 16 previously described obelisks found in seawater (Supplementary Table 4). We used two set of thresholds as species- and genus-level proxies (95% and 80% of identity) according to the consensus of uncultivated virus genome (UviG) criteria [14]. Out of the 55 total marine detected obelisks, 35 and 31 clusters at the species and genus level were found, respectively (see Supplementary Table 5), revealing an autochthonous diversity in marine ecosystems.

We also attempted to detect the Oblin-1 (~200 aa), the hallmark protein of the obelisks. To achieve this, we selected some of the obelisk-like hits obtained in the *Tara* Ocean transcriptomic dataset and predicted their ORFs and tertiary structures using ColabFold v1.5.5 (AlphaFold2 using MMseqs2) [15], which were compared with those obelisk sequences previously described [11]. The predicted Oblin-1 proteins from *Tara* Ocean samples were further confirmed showing a very similar 3D model, as observed in the former study [11] (Fig. 1B and C), indicating that Oblin proteins of the microbiome obelisks are structurally conserved in their marine relatives. Among the new detected obelisks in our study, some of them (*n* = 7) were found to encode a type III hammerhead ribozyme on each polarity. These self-cleaving motifs map in similar regions of the obelisk genomes, as previously described [11] (Fig. 1C, bottom left and right).

Very dissimilar obelisks were found in marine ecosystems at the nucleotide level. For instance the obelisk “contig SAMEA2619970_k119_37487” from the Arabian Sea yielded a nucleotide identity hit of only 28 bp (0.03% coverage) with other marine obelisks coinciding with the GYxDxG domain-A protein motif. Despite the lower identity and query coverage values, that sequence showed all the common hallmark features of obelisks (Fig. 1C, top left). Other examples of obelisk species were found in the Arabian Sea, Pacific, and Arctic Oceans (Fig. 1C). Furthermore, the predicted Oblins in the obelisks have a similar folding by means of high structural similarity score (Z) as those previously described [11] (Supplementary Fig. 1). We indeed detected obelisk-like sequences in the coldest seawater in Earth placed under the Antarctic Ross Ice Shelf; an extreme marine environment mainly driven by chemolithoautotrophy that host autochthonous polar viral assemblages [16, 17] (Supplementary Table 6). In our data mining of the *Tara* Ocean metatranscriptomes, we also used the Viroid Nominator tool for detecting de novo viroid-like elements as described [11], although no sequences with characteristics similar to obelisks were found in this study.

The relative abundance of obelisks was also estimated in *Tara* metatranscriptomic samples by fragment recruitment, as previously described in [18–20], with a threshold of query coverage ≥85% and nucleotide identity cutoff ≥95%. Results were normalized and expressed as kb recruited per kb of genome per Gb of metagenome (KPKG). To calibrate and provide a reference relative abundance, we also considered in our analysis those recently described uncultured, abundant RNA viruses (*n* = 5504) having RdRp from the oceanic RNA viruses catalog [6] (Supplementary Tables 7 and 8). Our results indicate that obelisks are surprisingly abundant in marine samples, in particular, at the deep chlorophyll maximum (DCM; Fig. 2). For instance, in the DCM Mediterranean Sea *Tara* sample SAMEA2619750, a cluster of four obelisks representing one species (SAMEA2620217_NODE_17961; highlighted in Fig. 2), such as obelisk SAMEA2620169_NODE_3959, reached a KPKG of 20, higher than the prokaryotic-fraction most abundant formerly described RNA viruses discovered in the *Tara* Oceans project [6]. Furthermore, these obelisks were widespread and abundant in many of the analyzed *Tara* samples. Other examples were the species SAMEA2623492_NODE_4235 and SAMEA2623516_NODE_10575 that showed a high abundance (8.3 KPKG) in our study at the DCM of the Gulf of Mexico. Furthermore, we also confirmed that these obelisks were fully recruited along the entire genome (e.g. abundant Obelisk_000080 in the Arabian and the Mediterranean Seas; Supplementary Fig. 2 and Fig. 2). These obelisks were not only detected in all temperate and tropical oceanic regions from all around the globe, but also in several stations of the *Tara* Artic Ocean dataset including samples from surface to mesopelagic (Fig. 1). For instance, Obelisk_001251 was only found in 13 prokaryotic *Tara* Arctic samples reaching an abundance up to 3.86 KPKG. Another obelisk found in the prokaryotic polar fraction was Arctic_ERR3586704_k119_355566 (Fig. 1C, top right; Supplementary Table 9).

**Figure 2:**
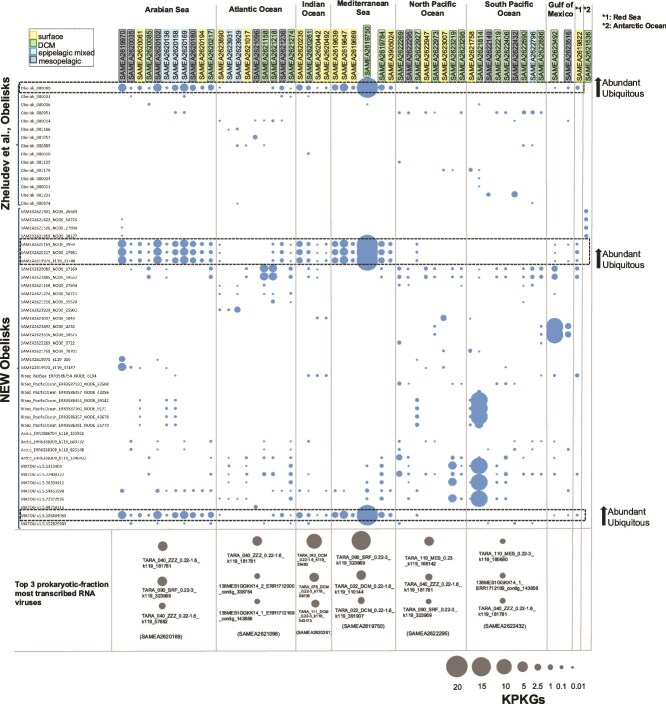
Relative abundance of marine obelisks estimated by transcriptomic fragment recruitment. Relative units are expressed as KPKG. Circle size represents the estimated KPKG. The y-axis displays the obelisk found, where the first 15 obelisk sequences belong to a previous study [11] and the rest are the potentially uncharacterized sequences of obelisks discovered here. The x-axis shows the different samples grouped by sea/ocean. To provide a reference for comparing obelisks abundance, a sample from each zone is selected at the bottom of the figure, where the relative abundance of marine RNA viruses in the prokaryotic fraction is also estimated showing the top 3 most abundant marine RNA viruses out of 5504 analyzed RNA viruses formerly described from the *Tara* Ocean project [6]. Colors in sample name of the horizontal axis depict the type of sample: surface, DCM, epipelagic mixed layer, or mesopelagic.

That the highest abundances were found at the DCM leads to the hypothesis that they might be associated with phytoplankton, such as cyanobacteria. However, CRISPR *in silico* massive search of viral protospacer match with a database of over 11 million of prokaryotic spacers [21] did not result in a successful identification of obelisks and potential hosts.

Finally, we explored the presence of obelisks in the eukaryotic metatranscriptomic fraction using the atlas of *Tara* Oceans Eukaryote Gene Catalog (the “MATOU version v1.5”) [22]. Data showed that some of these transcribed genes were in fact obelisks, or fractions of obelisks (Supplementary Table 10). A total of 8 obelisks were present in several samples corresponding to eukaryotic fractions (i.e., protists), and were distributed across all oceans and water depths. For example, MATOU-v1.5.105884968 and MATOU-v1.5.22908322 were present in 33 and 44 different samples, respectively (Supplementary Table 11), including polar stations. Other obelisks found in polar eukaryotic fractions were Arctic_ERR6338309_k119_1240422, Arctic_ERR6338309_k119_825148, and Arctic_ERR6338309_k119_683732 (Supplementary Table 9). Overall, obelisks in the eukaryotic fractions appear to be less abundant (KPKG values up to 0.48 in a DCM sample from the Mediterranean Sea (Supplementary Table 12)) than those obelisks found in the prokaryotic fraction reaching values up to 20 KPKG. Conversely, RNA viruses were previously found to be more abundant in the eukaryotic fractions than in the prokaryotic fraction of the *Tara* Ocean dataset [23]. Our data suggest that most of the obelisks found in our study would replicate in prokaryotes. We cannot rule out that the obelisks (*n* = 8) found in the eukaryotic fraction were sequenced from symbiotic bacteria associated with eukaryotic cells.

In conclusion, our data show that several obelisks are present and highly abundant in marine samples around the world. Our study highlights the ubiquity and abundance of obelisks, pointing to these viroid-like elements as significant, unrecognized components of marine biology, matching or exceeding the abundance of the most prevalent uncultured RNA viruses known to date in the prokaryotic fraction. These findings not only broaden our knowledge of unexpected subviral components in marine ecosystems but also emphasize the necessity for further research to gain a better understanding of their ecology, evolution, potential interactions with other marine organisms, and their contribution to the viral shunt.

## Supplementary Material

Supplementary_Material_wraf033

## Data Availability

The sequences of obelisks found in this study are available as supplementary material (SF1_TARA_NEW_OBELISKS.fasta). Supplementary Table 5 (sheet “80id”) shows the corresponding nomenclature of the obelisk discovered in this study with that proposed in the former obelisk study [11].
