# Supplementary material for: Viroid-like “obelisk” agents are widespread in the ocean and exceed the abundance of RNA viruses in the prokaryotic fraction
Source: ISME J. 2025 Feb 25;19(1):wraf033. doi: 10.1093/ismejo/wraf033 (PMC11922315; doi:10.1093/ismejo/wraf033)
Supplement: Supplementary_Material_wraf033 [file supplementary_material_wraf033.zip › Supplementary_Material_wraf033/Supplementary_Material_30-1.docx]

Supplementary Information

Viroid-like "Obelisk" agents are widespread in the ocean and exceed the abundance of RNA viruses in the prokaryotic fraction

Javier López-Simón^1,2^, Marcos de la Peña^3^, and Manuel Martínez-García^1,2*^

^1^Instituto Multidisciplinar para el Estudio del Medio Ramon Margalef, University of Alicante, Alicante, Spain.

^2^Departament of Phisiology, Genetics, and Microbiology, University of Alicante, Alicante, Spain

3Instituto de Biología Molecular y Celular de Plantas, Universidad Politécnica de Valencia-CSIC, Valencia, Spain

List of content:

1. Materials and Methods
2. Supplementary Tables
3. Supplementary Figures
4. Supplementary File
5. Supplementary data
6. References
7. **Materials and Methods**

***Data acquisition***.

The conservative database of 7,202 Obelisks used in this study was obtained from Zheludev et al. [1]

***Massive search of Obelisk-like elements in databases with Pebblescout***

A massive search of Obelisks was carried out using Pebblescout with default parameters, a recently released tool that allows very fast and efficient searches for subjects in petabase-scale nucleotide databases [2]. We ran the Obelisks database against the “Metagenomic” database, which includes all metagenomic and metatranscriptomic runs released in public SRA before the end of 2021.

***Detection of viroid-like Obelisk RNAs in transcriptomic datasets***

We analyzed a total of 159 marine metatranscriptomes from *Tara* Oceans [3] and a metatranscriptome of saline water under Ross Ice Shelf [4,5] searching for Obelisks using Blastn [6] and applying the following command: `blastn -outfmt "6 qseqid sseqid pident length mismatch gapopen qstart qend sstart send evalue bitscore qlen slen" -db Tara_metatranscriptome.fasta -query Obelisk_database.fasta -out Result.blast -evalue 0.00001`.We also used Viroid Nominator (VNom), a tool for nominating viroid-like de novo assembled contigs, to identify viroid-like sequences as described by Zheludev et al using the following parameters: `-max 2000-CF_k 10 -CF_simple 0 -CF_tandem 1 -USG_vs_all`. In the case of the seawater sample collected under Ross Ice Shelf, we performed a nucleotide Hidden Markov Model search (nHMMs) [7] against Obelisks database using hmmer-3.3.2 with the following parameters: `nhmmer --tblout RIS_Obelisk --cpu 30 Obelisk_database.fasta RIS_METATRANSCRIPTOMICS_MEGAHIT_assembly.fa`).

***Metatranscriptomics fragment recruitment analysis***

To estimate the relative abundance of Obelisks in *Tara* samples mainly in the prokaryotic fraction, we performed a metatranscriptome fragment recruitment. These analyses were carried out using stand-alone BLAST version 2.15.0 with the following command: `blastn -outfmt "6 qseqid sseqid pident length mismatch gapopen qstart qend sstart send evalue bitscore qlen slen" -db Obelisk_database.fasta -query Tara_metatranscriptome_reads.fasta -out Recruitment_obelisk_metat.blast -evalue 0.00001 -num_threads 30`. Resulting BLAST file was then analyzed by enveomics besthit2 [8] program for obtaining the best hit of each query for each subject. Then, by using R software, query coverage ≥85% and nucleotide identity cutoff ≥95% were applied and results were normalized according to metatranscriptome and genome size and expressed as kb of genome per Gb of metagenome (KPKG). For comparison, 5,504 marine RNA viruses obtained by Zayed et al 2022 were also included in our analysis.

***Ribozymes detection***

The detection of obelisks with ribozymes in assembled metatranscriptomes was carried out with the software Infernal ("INFERence of RNA ALignment") [9], using an obelisk-variant hammerhead type-III” ribozyme (ObV-HHR3) covariance model constructed by Zheludev et al. [1]. (/soft/infernal-1.1.5/src/cmsearch –tblout /scr/javier_scr/ERR3586920_hhr_cor.tbl hhr_cor.cm ERR3586920.fasta).

***RNA Secondary structures prediction***

Secondary RNA structures of Obelisks were predicted using RNAFold web server [10].

***Protein tertiary structure prediction***

Obelisk ORFs were predicted using Prodigal [11] and protein structure was predicted using ColabFold v1.5.5 (AlphaFold2 using MMseqs2)[12].

***Clustering of Obelisks***

Clustering into 95% identity “species-like” and 80% identity “genus-like” Obelisks was performed using *circuclust* ([https://github.com/rcedgar/*circuclust*](https://github.com/rcedgar/circuclust)) using the following parameters (circuclust -cluster Obelisk.fasta -id 0.95/0.8 -fastaout Obelisk_centroids.fa -tsvout Circuclust_clustering_95/80id.tsv)

***Comparison of tertiary structure fold of Oblin-1***

The comparison of the tertiary structure of the oblins represented in Supplementary Figure 1 was carried out using two programs, DALI web server [13] to get the structural similarity score (Z) and ChimeraX [14] for the 3D model of the overlapping structures.

1. **Supplementary tables**

**Supplementary Table ST1** | Results of the search of the Obelisks database against Pebblescout “Metagenomic” database (>80 PBSscore). This table is provided as a separate file <ST1_Pebblescout_Obelisks_results.xlsx >.

**Supplementary Table ST2** | *Tara* Oceans metatranscriptomes analyzed in this study. This table is provided as a separate file < ST2_Analyzed_TARA_metatranscriptomes.xlsx>.

**Supplementary Table ST3** | Results of the search of Obelisks in *Tara* metatranscriptomics using BLASTn (>70% identity, >50% coverage). This table is provided as a separate file < ST3_TARA_Metat_Obelisk_BLAST+70id_50cov.xlsx>.

**Supplementary Table ST4** | Novel Obelisks discovered in this study along with the description of the sample from which it was obtained and the search technique. This table is provided as a separate file < ST4_Info_New_Obelisks.xlsx>.

**Supplementary Table ST5** | Circlust clustering into 95% identity “species-like” and 80% identity “genus-like” of the 40 novel Obelisk genomes discovered in this study. Furthermore, this table showed the corresponding Obelisk nomenclature following that proposed by Zheludev et al. (2024); see sheet named as “80id”. This table is provided as a separate file < ST5_Obelisk_clustering.xlsx>.

**Supplementary Table ST6** | Results of the search of Obelisks in marine water under Ross ice shelf sample using Hidden Markov Models. (e-value <0.01).

**Supplementary Table ST7** | Relative abundance of marine Obelisks estimated by transcriptomic fragment recruitment in each marine sample. This table is provided as a separate file < ST7_Relative_Abundance_Obelisk_Tara.xlsx >.

**Supplementary Table ST8** | Relative abundance of RNA viruses catalog in one sample of each sea/ocean estimated by transcriptomic fragment recruitment. This table is provided as a separate file < ST8_Relative_Abundances_RNASullivan >.

**Supplementary Table ST9** | Results of the search of Obelisks in Arctic Samples. In the first Excel sheet, Obelisks hits against Tara Arctic samples using Pebblescout (50 PBSscore) are shown. In the second sheet, BLASTn results of three assembled Tara Arctic samples against Obelisk database. This table is provided as a separate file < ST9_Arctic_Obelisk.xlsx>.

**Supplementary Table ST10** | Results of the search of Obelisks in the Tara Oceans Eukaryote Gene Catalog (the "MATOU") version 1.5 using BLAST.

**Supplementary Table ST11** | Results for the search of the Obelisks detected in the MATOU metatranscriptomic occurrences file. The table contains the name of the Obelisks, the samples in which each Obelisk was found and its abundance in RPKM (reads per kilo base covered per million of mapped reads). This table is provided as a separate file < ST11_Obelisk_MATOU_occurrences.xlsx>.

**Supplementary Table ST12** | Relative abundance of Obelisks estimated by transcriptomic fragment recruitment in 14 *Tara* samples corresponding to size fractions for protists. This table is provided as a separate file < ST11_Relative_Abundance_Obelisk_Tara_Eukaryotic.xlsx >.

1. **Supplementary Figures**

**
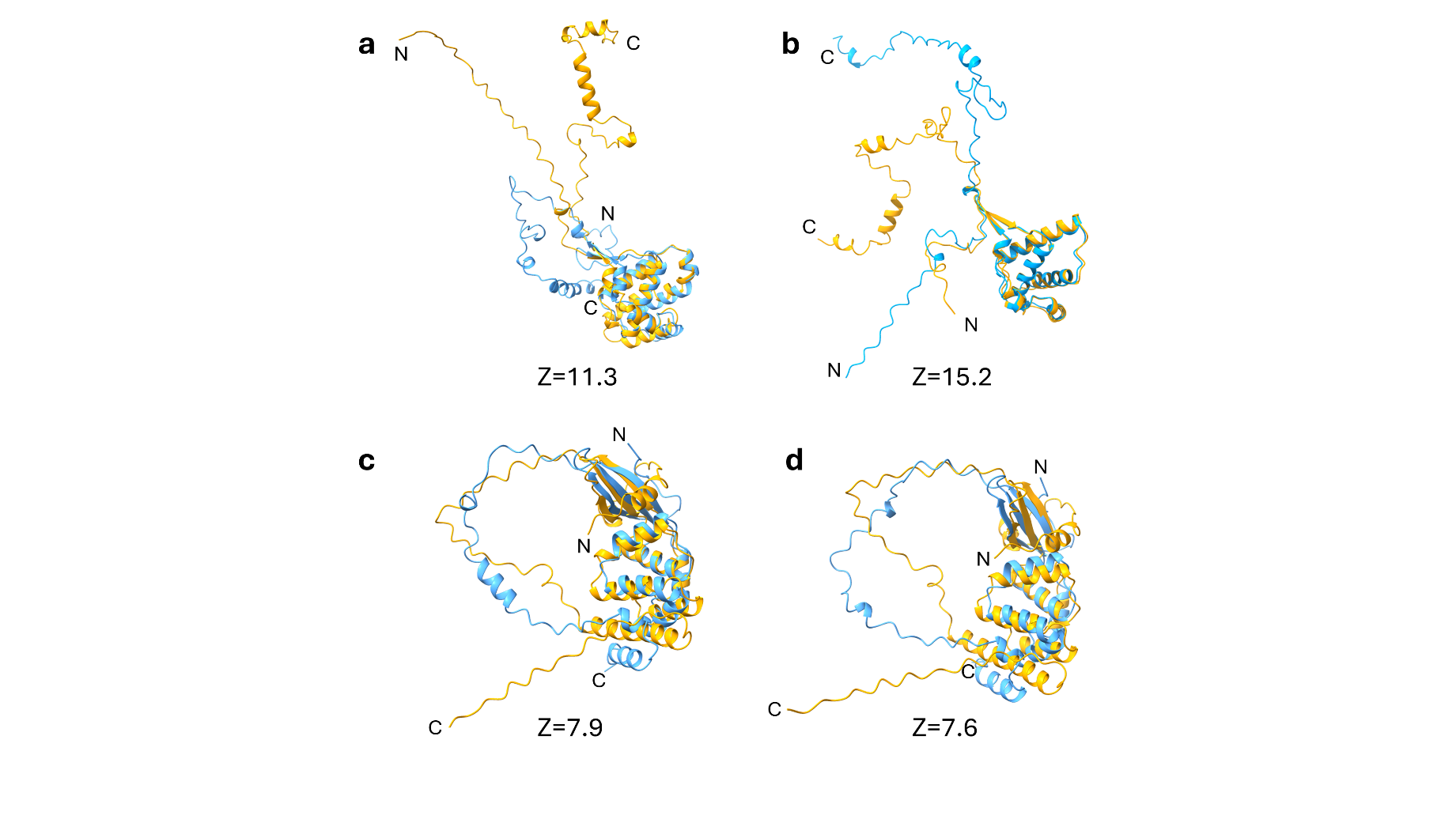
**

**Supplementary Figure 1** | Representation of the fold comparison of four Oblin-1 from novel Obelisks versus Oblin-1 from previously described Obelisks. Structural similarity score (Z) calculated by DALI web server is represented and C-terminal and N-terminal of each protein chain is also depicted. **(a)** SAMEA2619970_k119_37487 Oblin-1 (blue) and Obelisk_001179 Oblin-1 (yellow) **(b)** Arctic_ERR3586704_k119_355566 Oblin-1 (blue) and Obelisk_001166 Oblin-1 (yellow) **(c)** Riboz_PacificOcean_ERR3586957_NODE_43256 Oblin-1 (blue) and Obelisk_000836 Oblin-1 (yellow) **(d)** Riboz_PacificOcean_ERR3587060_NODE_9171 Oblin-1 (blue) and and Obelisk_000836 Oblin-1 (yellow).


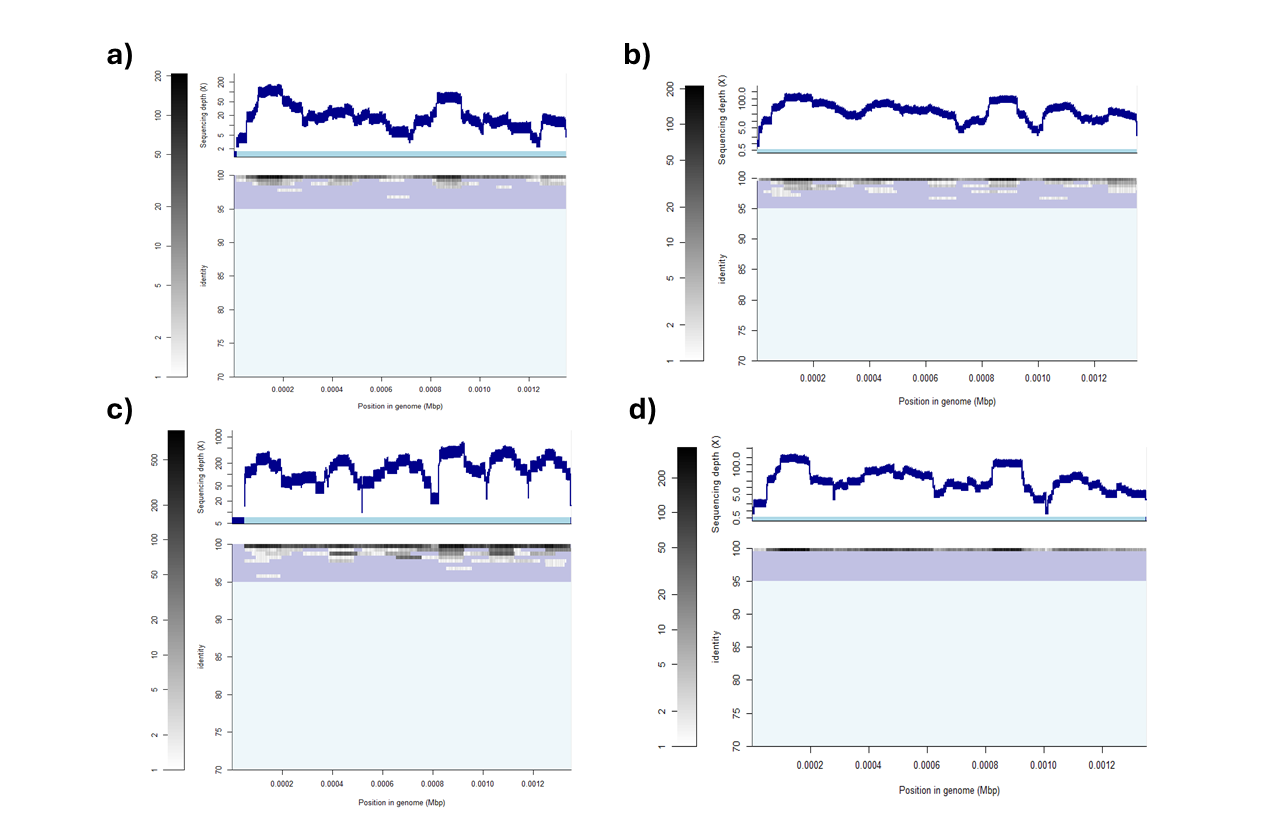


**Supplementary Figure 2** | Metatranscriptomic fragment recruitment of Obelisk_000080_000002_000001 in marine samples **(a)** SAMEA2620169 (Arabian sea), **(b)** SAMEA2620102 (Arabian sea), **(c)** SAMEA2619970 (Arabian sea) and **(d)** SAMEA2619750 (Mediterranean sea). The Obelisk is fully recruited with reads at 95–100% identity.

1. **Supplementary Data**

There is supplementary online data, such as the FASTA file of obelisk genomes

1. **Supplementary File**

**Supplementary File 1** | Fasta nucleotide file with the sequences of new Obelisks found in this study < SF1_TARA_NEW_OBELISKS.fasta>.

1. **References**
2. Zheludev IN, Edgar RC, Lopez-Galiano MJ, de la Peña M, Babaian A, Bhatt AS, et al. Viroid-like colonists of human microbiomes. Cell. 2024;187(23):6521-6536.e18. doi:10.1016/j.cell.2024.09.033
3. Shiryev SA, Agarwala R. Indexing and searching petabase-scale nucleotide resources. Nat Methods. 2024;21(6):994-1002. doi:10.1038/s41592-024-02280-z
4. Pesant S, Not F, Picheral M, Kandels-Lewis S, Le Bescot N, Gorsky G, et al. Open science resources for the discovery and analysis of Tara Oceans data. Sci Data. 2015;2(1):150023. doi:10.1038/sdata.2015.23
5. Martínez-Pérez C, Greening C, Bay SK, Lappan RJ, Zhao Z, De Corte D, et al. Phylogenetically and functionally diverse microorganisms reside under the Ross Ice Shelf. Nat Commun. 2022;13(1):117. doi:10.1038/s41467-021-27769-5
6. Lopez-Simon J, Vila-Nistal M, Rosenova A, De Corte D, Baltar F, Martinez-Garcia M. Viruses under the Antarctic Ice Shelf are active and potentially involved in global nutrient cycles. Nat Commun. 2023;14(1):8295. doi:10.1038/s41467-023-44028-x
7. Altschul SF, Gish W, Miller W, Myers EW, Lipman DJ. Basic local alignment search tool. J Mol Biol. 1990;215(3):403-10. doi:10.1016/S0022-2836(05)80360-2
8. Eddy SR. Accelerated Profile HMM Searches. PLoS Comput Biol. 2011;7(10):e1002195. doi:10.1371/journal.pcbi.1002195
9. Rodriguez-R LM, Konstantinidis KT. The enveomics collection: a toolbox for specialized analyses of microbial genomes and metagenomes. PeerJ Prepr. 2016;4:e1900v1. doi:10.7287/peerj.preprints.1900v1
10. Nawrocki EP, Eddy SR. Infernal 1.1: 100-fold faster RNA homology searches. Bioinformatics. 2013;29(22):2933-2935. doi:10.1093/bioinformatics/btt509
11. Lorenz R, Bernhart SH, Höner zu Siederdissen C, Tafer H, Flamm C, Stadler PF, et al. ViennaRNA Package 2.0. Algorithms Mol Biol. 2011;6(1):26. doi:10.1186/1748-7188-6-26
12. Hyatt D, Chen GL, LoCascio PF, Land ML, Larimer FW, Hauser LJ. Prodigal: prokaryotic gene recognition and translation initiation site identification. BMC Bioinformatics. 2010;11(1):119. doi:10.1186/1471-2105-11-119
13. Mirdita M, Schütze K, Moriwaki Y, Heo L, Ovchinnikov S, Steinegger M. ColabFold: making protein folding accessible to all. Nat Methods. 2022;19(6):679-682. doi:10.1038/s41592-022-01488-1
14. Holm L, Laiho A, Törönen P, Salgado M. DALI shines a light on remote homologs: One hundred discoveries. Protein Sci. 2023;32(1):e4519. doi:10.1002/pro.4519
15. Meng EC, Goddard TD, Pettersen EF, Couch GS, Pearson ZJ, Morris JH et al. UCSF ChimeraX: Tools for structure building and analysis. Protein Sci. 2023;32(11):e4792. doi:10.1002/pro.4792
